# Draft Genome Sequence of the Wood-Staining Ascomycete *Chlorociboria aeruginascens* DSM 107184

**DOI:** 10.1128/MRA.00249-19

**Published:** 2019-04-25

**Authors:** Enrico Büttner, Christiane Liers, Anna Maria Gebauer, Jérôme Collemare, Jorge Carlos Navarro-Muñoz, Martin Hofrichter, Harald Kellner

**Affiliations:** a Department of Bio- and Environmental Sciences, International Institute Zittau, Technische Universität Dresden, Zittau, Germany; b Westerdijk Fungal Biodiversity Institute, Utrecht, The Netherlands; University of California, Riverside

## Abstract

Chlorociboria aeruginascens DSM 107184 is a wood-decomposing ascomycetous fungus known to produce the bluish-green dimeric naphthoquinone derivate xylindein. Here, we present the first draft genome sequence, which contains 588 contigs with a total length of 33.1 Mb. Altogether, 8,648 protein-coding genes were predicted.

## Abstract (precis)

Chlorociboria aeruginascens DSM 107184 is a wood-decomposing ascomycetous fungus known to produce the bluish-green dimeric naphthoquinone derivate xylindein. Here, we present the first draft genome sequence, which contains 588 contigs with a total length of 33.1 Mb.

## ANNOUNCEMENT

The bluish-green-colored ascomycete Chlorociboria aeruginascens belongs to the Helotiaceae family. It is well known for its characteristic spalting of infected wood during deadwood decomposition. C. aeruginascens is a sequential wood decomposer, i.e., a soft rot fungus, colonizing different deciduous tree species (hardwood), with a worldwide distribution (1). Chemical derivatization and spectroscopic analyses of the bluish-green pigment xylindein revealed its absolute configuration and its tautomeric structure as a dimeric naphthoquinone (2), and most of its total synthesis has been reported (3). The chemical characteristics of xylindein, like stability or insolubility, in common organic solvents, as well as its electronic properties (4), make xylindein an interesting subject of biotechnological research. Bluish-green wood has been utilized for artistic purposes since the 15th century (5, 6). However, large-scale production of the pigment using *C. aeruginascens* is difficult due to slow growth and difficult handling of the fungus (1, 7).

The present draft genome sequence of *C. aeruginascens* will help identify the genes coding for proteins involved in the biosynthesis of the pigment xylindein and for extracellular enzymes involved in the decomposition of the lignocellulosic complex.

The strain DSM 107184 (ribosomal cistron GenBank accession number MK480517) was isolated from a fruiting body growing on Fagus sylvatica deadwood (Lackenwald Weyer, Austria; 47°26′24.0″N, 14°18′00.0″E). Mycelium was obtained from 6-week-old malt agar plates (2.5% apple peel). Afterwards, biomass was scraped off, freeze dried, and used to extract the genomic DNA by a standard cetyltrimethylammonium bromide (CTAB)-based method. Genomic DNA was sonographically sheared (S2 ultrasonicator; Covaris, Woburn, MA, USA), and a 200-bp library was constructed using the Ion Plus fragment library kit (Thermo Fisher, Darmstadt, Germany). The genome was sequenced on an Ion Torrent personal genome machine (PGM) using the Ion PGM sequencing 200 kit version 2 and a 318v2 Chip (Thermo Fisher). The resulting 5.9 million reads were filtered to include only lengths of 160 to 280 bp and were assembled using MIRA 4.0 (8) first and Geneious R11 (9) after (parameter highest sensitivity/slow) to join overlapping contigs and to filter for duplicate contigs. The assembly contains 588 contigs (maximum length, 454,753 bp; *N*_50_ value, 110,634) (10) with a total length of 33.1 Mb and a G+C content of 43.1%. AUGUSTUS version 3.2.2 (11) and the predictor set to Coccidioides immitis were used to predict 8,648 protein-coding genes. Quantitative genome statistics were analyzed using BUSCO version 3 (12, 13) (fungal data set Ascomycota_*odb9*), which reported a genome completeness of 98.0% (complete BUSCOs). Specific enzymes, like lignocellulolytic hydrolases and oxidoreductases (Table 1), were annotated and filtered using Blast2GO version 5.2.2 (BioBam, Valencia, Spain) or identified in the genome using BLASTP searches (BLOSUM62 matrix; E value, 1e^−1^) with known crystal structure-based reference sequences (RCSB PDB). Prediction of carbohydrate-active enzymes (CAZymes) using dbCAN (14) (E value, <1e^−15^; coverage, >0.35) resulted in 497 identified genes (Table 1), among them those encoding enzymes that act on aromatic substrates. Secondary metabolite (SM) biosynthetic gene clusters (BGCs) were predicted using antiSMASH version 4.1.0 (15). A total of 32 BGCs were identified, including BGCs for the production of 14 polyketides, four nonribosomal peptides, one hybrid polyketide-nonribosomal peptide, five terpenes, and eight nonribosomal peptide-like SMs. One of the polyketide BGCs likely controls the production of xylindein.

**TABLE 1:** CAZyme classes and enzymes of interest detected in the genome of DSM 107184

Enzyme or domain group^*a*^	No. of proteins	GenPept accession no.
Glycoside hydrolases	219	
Glycosyltransferases	92	
Polysaccharide lyases	1	
Carbohydrate esterases	66	
AA	72	
Associated modules		
CBM	47	
Cellulose-binding domain CBM1	23	
Enzymes of interest		
Unspecific peroxygenase	1^*b*^	TAQ89918
Cytochrome P450 enzymes	55	
Laccase	13	
Generic peroxidase (class II-related)	1	TAQ86696
Dye-decolorizing peroxidase	1	TAQ91334
Catechol 2,3-dioxygenase	1	TAQ86074

a AA, auxiliary activity; CBM, carbohydrate-binding modules.

b Chloroperoxidase-like superfamily, Pfam PF01328, Peroxidase_2.

### Data availability.

This whole-genome shotgun project has been deposited at DDBJ/ENA/GenBank under the accession number NCSK00000000. The version described in this paper is version NCSK02000000. The Sequence Read Archive (SRA) accession number is SRR5435769, associated with the BioProject number PRJNA382475.
